# Recent Progress in Infant Welfare Work

**Published:** 1914-07

**Authors:** 


**Recent Progress in Infant Welfare Work.** van Ingen, in the American Journal of Diseases of Children, June, 1914, gives a survey of this work at home and abroad which should be widely read by everybody, not physicians only, as we can tell by comparing our work with that of others if we are doing our full duty towards the most helpless part of humanity. It will act as a guide for anybody who wants to engage in these meritorious activities.—C. G. L-W.

